# Central odontogenic fibroma: Retrospective study of 8 clinical cases

**DOI:** 10.4317/medoral.17129

**Published:** 2011-12-06

**Authors:** Radia Hrichi, Jordi Gargallo-Albiol, Leonardo Berini-Aytés, Cosme Gay-Escoda

**Affiliations:** 1 DDS. Fellow of Oral Surgery and Implantology, School of Dentistry. University of Barcelona (Spain); 2 DDS, PhD. Professor of the Master of Oral Surgery and Implantology. School of Dentistry. University of Barcelona (Spain). Researcher of the IDIBELL Institute; 3 DDS, MD, PhD. Professor of Oral and Maxillofacial Surgery. Professor of the Master of Oral Surgery and Implantology. School of Dentistry, University of Barcelona (Spain). Researcher of the IDIBELL Institute; 4 DDS, MD, PhD. Chairman and Professor of Oral and Maxillofacial Surgery. Director of the Master of Oral Surgery and Implantology. School of Dentistry, University of Barcelona. Coordinator/Researcher of the IDIBELL Institute. Oral and Maxillofaxial Surgeon of the Teknon Medical Center, Barcelona (Spain)

## Abstract

Introduction and Objectives: The central odontogenic fibroma (COF) is a benign odontogenic tumour derived from the dental mesenchymal tissues. It is a rare tumour and only 70 cases of it have been published. Bearing in mind the rareness of the tumour, 8 new cases of central odontogenic fibroma have been found by analyzing the clinical, radiological and histopathological characteristics of COF. Patients and Method: A retrospective study was carried out on 3011 biopsies in the Service of Oral and Maxillofacial Surgery of the Dental Clinic of Barcelona University between January 1995 and March 2008. 85 odontogenic tumours were diagnosed of which 8 were central odontogenic fibroma. The radiological study was based on orthopantomographs, periapical and occlusal radiographies and computerised tomographics. The variables collected were: sex, age, clinical characteristics of the lesion, treatment received and possible reappearances of the tumour. Results: The central odontogenic fibroma represents 9.4% of all odontogenic tumours. Of the 8 cases, 5 were diagnosed in men and 3 in women. The average age was 19.9 years with an age range of 11 to 38 years. The most common location of the tumour was in the mandible. All cases were associated with unerupted teeth. Of the 8 tumours, 3 provoked rhizolysis of the adjacent teeth and 4 cases caused cortical bone expansion. 50% of the patients complained of pain associated to the lesion. No case of recurrence was recorded up to 2 years after the treatment. Conclusions: Central odontogenic fibromas usually evolve asymptomatically although they can manifest very aggressively provoking dental displacement and rhizolysis. Radiologically, COF manifest as a uni or multilocular radiotransparent image although they can be indistinguishable from other radiotransparent lesions making diagnosis more difficult. COF treatment involves conservative surgery as well as follow-up patient checks.

** Key words:** Odontogenic tumour, central odontogenic fibroma.

## Introduction

Odontogenic tumours make up a complex group of maxillary lesions derived from the dental mesenchymal tissues. This pathology represents 2.8% of the biopsies carried out by the Service of Oral and Maxillofacial Surgery of the Dental Clinic of Barcelona University between January 1995 and March 2008, giving us an idea of its rarity. However, the clinical and histopathological distinctive characteristics of these tumours as well as being a specific and unique pathology of the maxillary make them an important and interesting case study.

Central odontogenic fibroma (COF) is a rare odontogenic tumour made up of dental mesenchymal tissues (1,2). Since being introduced into the World Health Organisation (WHO) classification of odontogenic tumours in 1971, COF has caused confusion due to its nature and definition. This means that there is little reliable data regarding its relative occurrence. COF was considered to be one of the most frequent odontogenic tumours for a long time, with an incidence of 23%. This confusion was due to the fact that a hyperplasm of the dental follicle was diagnosed as COF because of the histological similarities between both clinical entities (2). In 1980, Gardner (1) attempted to investigate further into the clarification of lesions previously diagnosed as COF. He defined two histological variants. First, a hyperplastic dental follicle with a connective fibrous tissue and small amounts of odontogenic epithelium and a WHO type or complex with connective cellular tissue, a prominent epithelial component and the presence of variable quantities of dentine or cement-like tissue. The complex type, mainly found in the mandible, was usually more aggressive, provoking expansions of the cortical bones, paresthesia of the interior dental nerve and pain (3,4). COF of the granular cell type constitutes a histological variant with multiple lobules of granular cells within dense nests and/or strands of apparently inactive odontogenic epithelium islets (4-6). Granular cells can be detected in numerous tumours such as ameloblastoma, ameloblastic fibroma and COF. Sometimes, these cells predominate in the tumour as in the case of the ameloblastic fibroma of granular cells or the odontogenic fibroma of granular cells. In 2003, Piattelli et al (6) documented the only case of a malignant odontogenic tumour of granular cells which was clinically aggressive, called an odontogenic sarcoma of granular cells. This tumour was identified in a 40 year old woman that complained of a painful lesion in the upper right part of the maxillary. The tumour had been growing for 3 months and was invading the maxillary cavity. An initial treatment with hemimaxilectomy of the affected area failed since at 16 months a recurrence formed mainly of malignant granular cells had invaded the condyle and upper right section of the mandible. These researchers suggested that the presence of granular cells increased the risk of the tumour being malignant.

COF is a slow and persistent growing tumour and is more frequently found in women. Clinically, it tends to manifest as an asymptomatic swelling although it can appear in a more aggressive way provoking dental displacement and rhizolysis (5,6). Histologically, it is defined as a fibroplastic neoplasia that contains inactive odontogenic epithelium and variable quantities of calcified material (2). COF can be found to be associated with the crown of an included tooth or the roots of erupted teeth. The upper jaw shows a predilection for the front part of the mouth whilst in the mandible there is a predilection for the back part of the mouth (2,3). Radiologically, COF manifests as a uni or multilocular image with well defined margins and surrounded by a sclerotic halo. The multilocular radiotransparent form is more frequently associated with complications such as severe reabsorption of the roots of adjacent teeth, displacement of near-lying teeth or some included tooth (2,7). 

The treatment of COF involves conservative surgery through enucleation of the lesion and the use of a curette to heal the remaining cavity. The clinical cases and the series of published cases to date have contributed to knowledge about clinical and histological characteristics of COF. Despite this, certain gaps in knowledge remain with regards to the definition and etiology of this entity such as reasons for COF recurrence following surgical treatment since some cases have been recorded. For this reason, we decided to carry out a study using 8 new COF cases analyzing the clinical, radiological and histopathological characteristics of this tumour. 

## Patient and Methods

A retrospective study of 3011 biopsies was carried out in the Service of Oral and Maxillofacial Surgery of the Dental Clinic of Barcelona University between January 1995 and March 2008. 

85 odontogenic tumours were diagnosed, 8 of which were central odontogenic fibroma. The radiological study was based on orthopantomographics, periapical and occlusal radiographies and computerised tomography. The variables collected were: sex, age, clinical characteristics of the lesion, treatment received and possible recurrences. The data collected was processed with version 12.0 of the Statistical Package for Social Science (SPSS; Chicago, USA. License of Barcelona University).

## Results

COF represents 9.4% of odontogenic tumours (0.003% of the total biopsies carried out over the 13 year period). It occupies the fourth highest position in order of tumour frequency; the most common tumour was the odontoma ( Table 1). 

There was a slight predilection for the male sex (1,67:1) and the average age was 19.9 years with an age range of 11 to 38 years. All cases were associated with unerupted teeth. 

**Figure 1:**
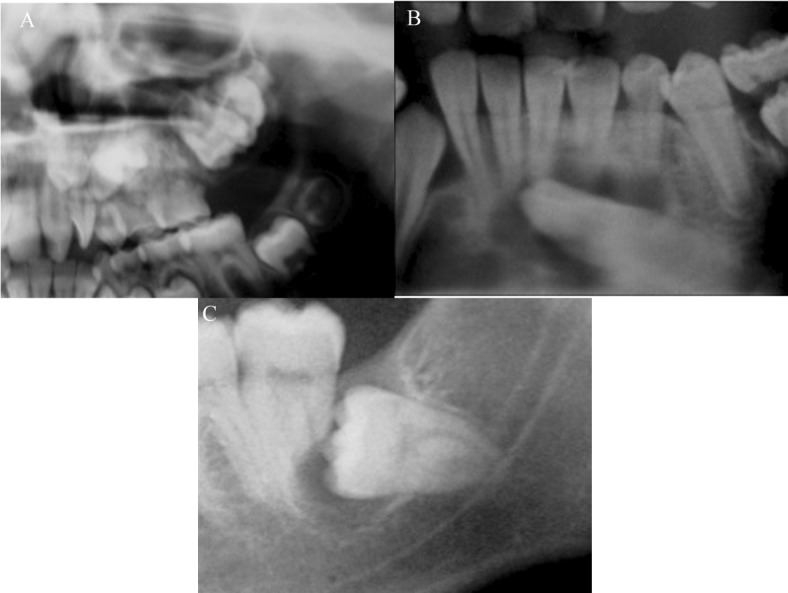
Orthopantomografic views. A) Orthopantomografic view of the case 5. B) Orthopantomografic view of the case 7. C) Orthopantomografic view of the case 8.

**Figure 2:**
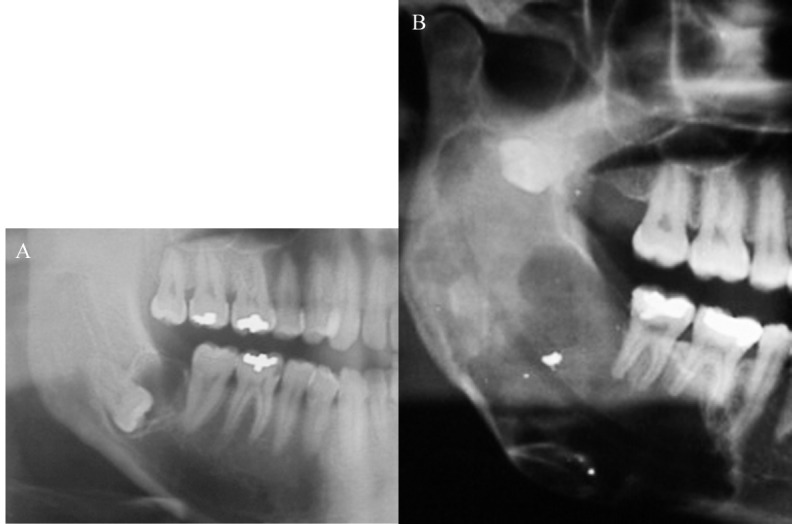
Orthopantomografic views. A) Orthopantomografic view of the case 2. B) Orthopantomografic view of the case 4.

The most common location of the tumour was in the mandible, in 75% of the cases. Of 6 COF cases in the mandible, 5 were associated with a third molar included (Figs. 1.C, 2.A,B) and one case with a canine tooth included (Fig. 1.A). The 2 cases recorded in the upper jaw were situated in the posterior zone. Radiologically, 5 cases appeared as unilocular radiotransparent images with well-defined margins; one case showed a multilocular radiotransparent image (Fig. 2.B); and another case, corresponding to an 11-year old patient showed a mixed image with badly defined margins that displaced the bacteria 2.4 and 2.5 to a more apical position (Fig. 1.B). 

**Figure 3:**
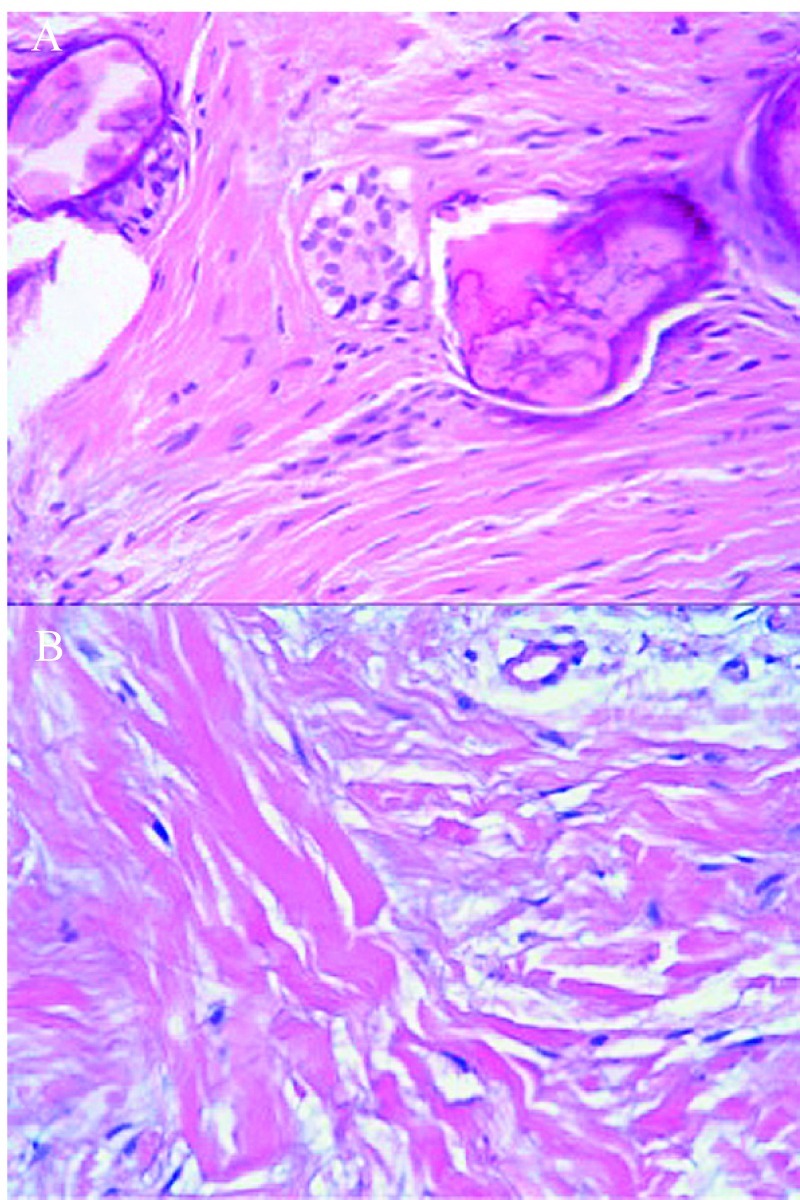
Histopathologic features. A) Histopathologic features of the case 5: Epithelial island in a stroma fbroblastic cells with calcifid material (x 400). B) Histopathologic features of the case 7: Presence of fibroblastic proliferation and dense collagen stroma (x 400).

The histological study showed in all cases a fibroblastic neoplasm confirming the diagnosis of FOC (Fig. 3).

As regards the clinical manifestations of the 8 tumours, 3 provoked rhizolysis of the adjacent teeth (Figs. 1.B,C, 2.B) and 4 caused expansion of the cortical bones. 50% of the patients complained of slight pain around the area of the lesion. In all cases, the treatment was conservative surgery through the enucleation of the lesion and the use of a curette to heal the remaining cavity. No case of recurrence was recorded up to 2 years later. In  Table 2, the clinical and radiological characteristics of each case are summarised.

**Table 1:**
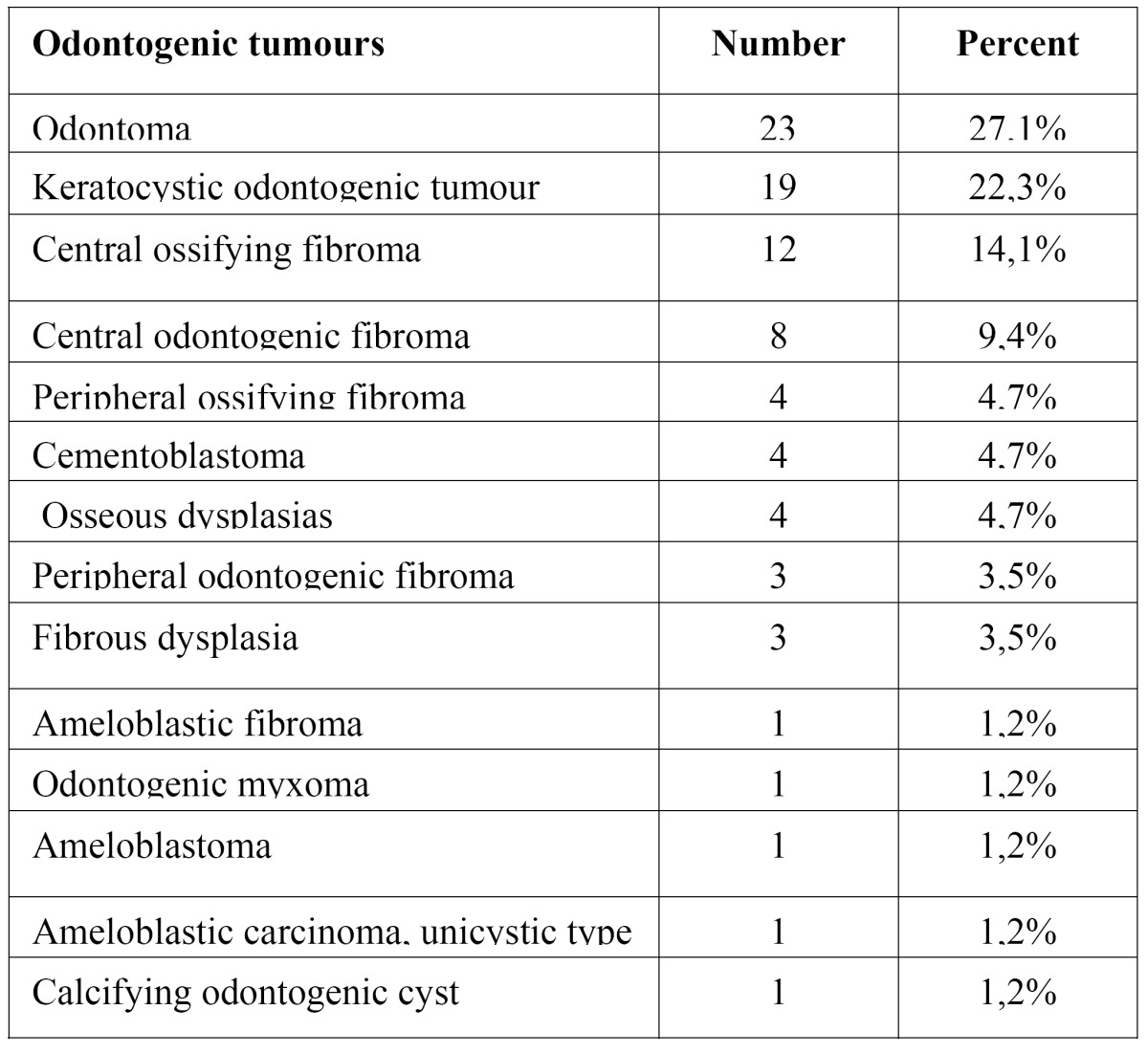
Incidence of odontogenic tumours between January 1995 and March 2008.

**Table 2:**
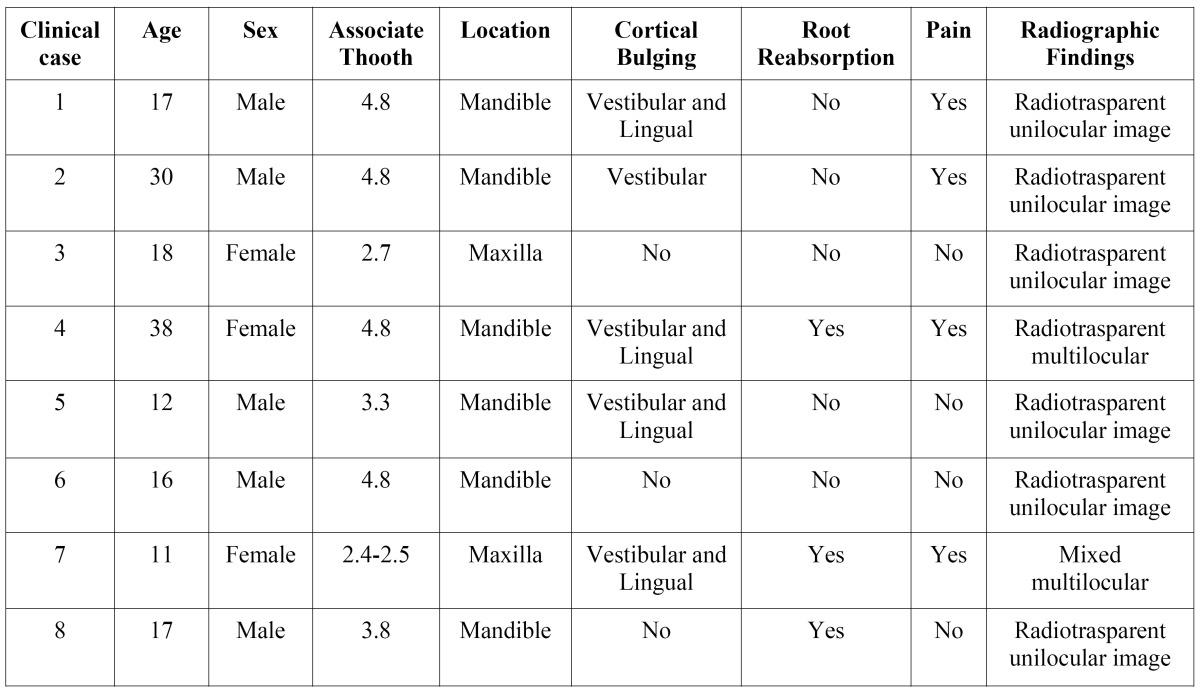
Clinical and radiologic features of the 8 clinical cases.

## Discussion

COF is generally diagnosed in patients in the second and third decade of their life although cases have been recorded of patients between the ages of 5 and 80 years (3). Our sample does not fully agree with this assertion since there is a prevalent predominance of patients diagnosed between 10 and 30 years of age (75%).

Another interesting epidemiological aspect is the distribution by sex. The majority of former studies reported a predilection of COF in the female sex; however, in our study, 62.5% of the patients were male. This matches up with the study of Buchner et al in which 62% of the patients were male (2). In a revision of 47 COF cases, Bueno et al (3) found that the most common location of the tumour was in the upper jaw with 55%. They observed a predilection for the frontal region of the upper jaw whilst in the mandible there was a predilection for the posterior region. In our study, the most frequent location is the mandible in 75% of the cases of which 83% included a third molar. The two cases recorded in the upper jaw were found in the posterior zone. This coincides with Svirsky et al (8) who analyzed 15 cases of COF and reported that 80% occurred in the mandible. In the revision carried out by Handlers et al (9), of the 39 cases of COF found, 56% were reported to have occurred in the upper jaw and 44% in the mandible whith predilection of COF in the female sex (3:1). The lastest revision in the literature by Ramer et al (10), reported a similar incidence in the maxilla and mandible (1:1) with 69% of cases found in women (47:68).

Radiologically, the images obtained in the majority of our cases do not significantly differ from the clasical descriptions: radiolucent image, usually unilocular well-defined borders.

In one cas, there was a multilocular radiotransparent image of 4.5 x 6cm that extended from the 4.6 zone to the coronoid apophysis in relation to the 4.8 included displaced to this position (case nº4). In another case, an 11-year old patient, the tumour showed a mixed, multilocular image with badly defined margins in the second quadrant which caused the displacement of the tooth germ 2.4 and 2.5 to a more apical position (case nº7). In their revision of COF, Bueno et al (3) reported only 2 cases that showed a mixed radiological image. 

Coinciding with Daniels et al (11), we observed that small sized lesions tended to be unilocular where as the larger lesions were multilocular and showed more aggressive behaviour provoking complications such as severe reabsorption of the roots of adjacent teeth, radicular displacement of adjacent teeth or an included tooth and expansion of the cortical bones.

Clinically, the majority of COF evolved in an asymptomatic manner causing a slow expansion of the cortical bones (3). The clinical signs observed in the cases described here are from the greatest to the least frequency: prominence of the vestibular cortical and/or lingual (75%), pain (50%) and rhizolysis (37.5%). As mentioned earlier, in our study we observed that the multilocular and mixed forms behaved more aggressively.

The great variability of clinical and radiological characteristics that COF can present oblige us to incorporate numerous nosological entities in the differential diagnosis such as the ameloblastoma, uniquistic ameloblastomas, calcifying odontogenic cysts, COF, follicular cysts and other entities that manifest radiologically as a radiotransparent image (12). The correct diagnosis will be determined by the histological study of the lesion. Covani y cols. in an article published in 2005 presented a case of COF associated with the root of an erupted tooth. The authors argue the importance of making a correct differential diagnosis of COF with endodontic lesions showing the same image radiological lucent image (13). The diagnosis of the FOC is determined by the histology of the lesion.

The treatment of COF is conservative surgery through the enucleation of the lesion and the use of a curette to heal the remaining cavity. Recurrences of COF are not common. In our study, no case of recurrence has been reported in 5 years of tracking. Dunlap and Barker (14) presented 2 cases of COF in the upper jaw treated with the enucleation of the lesion and the use of a curette to heal the remaining cavity with a consequent 9 year tracking period without evidence of COF recurrence. In their revision of 68 cases of COF, Ramer et al (10) reported 5 cases of recurrence and suggested that the cases of recurrence are not related to the histological type but due to an incomplete surgical removal of the lesion. In 2004, Alawi et al (15) published a recurring case of COF in the form of an ameloblastoma in a 65-year old male which presented a mixed image in the right posterior part of the mandible. The lesion reappeared 4 years later in the right part of the mandible. The researchers suggested that the recurrence was due to an incomplete removal of the initial tumour since it was not encapsulated and presented a close connection to the interior dental nerve which made its correct excision more difficult. Despite the low recurrence rate, a post-operation tracking should be carried out on the patient up to at least 5 years after surgical intervention.

## References

[1] Gardner DG (1980). The central odontogenic fibroma: an attempt at clari¬fication. Oral Surg Oral Med Oral Pathol.

[2] Buchner A, Merrell PW, Carpenter WM (2006). Relative frequency of central odontogenic tumors: a study of 1,088 cases from Northern California and comparison to studiesfrom other parts of the world. J Oral Maxillofac Surg.

[3] Bueno S, Berini L, Gay C (1999). Central odontogenic fibroma: a review of the literature and report of a new case. Med Oral.

[4] Cercadillo-Ibarguren I, Berini-Aytés L, Marco-Molina V, Gay-Es¬coda C (2006). Locally aggressive central odontogenic fibroma associated to an inflammatory cyst: aclinical, histological and immunohistoche¬mical study. J Oral Pathol Med.

[5] Reichart P, Philipsen HP, Moegelin A, Thalmann U (2006). Central odon¬togenic fibroma, granular cell variant. Oral Oncol.

[6] Piattelli A, Rubini C, Goteri G, Fioroni M, Maiorano E (2003). Central granular cell odontogenic tumour: report of the first malignant case and review of theliterature. Oral Oncol.

[7] Kaffe I, Buchner A (1994). Radiologic features of central odontogenic fibroma. Oral Surg Oral Med Oral Pathol.

[8] Svirsky JA, Abbey LM, Kaugars GE (1986). A clinical review of central odontogenic fibroma: with the addition of three new cases. J Oral Med.

[9] Handlers JP, Abrams AM, Melrose RJ, Danforth R (1991). Central odon¬togenic fibroma: clinicopathologic features of 19 cases and review of the literature. J OralMaxillofac Surg.

[10] Ramer M, Buonocore P, Krost B (2002). Central odontogenic fibroma--report of a case and review of the literature. Periodontal Clin Investig.

[11] Daniels JS (2004). Central odontogenic fibroma of mandible: a case re¬port and review of the literature. Oral Surg Oral Med Oral Pathol Oral Radiol Endod.

[12] Sadeghi EM, Sewall SR, Dohse A, Novak TS (1995). Odontogenic tu¬mors that mimic a dentigerous cyst. Compend Contin Educ Dent.

[13] Covani U, Crespi R, Perrini N, Barone A (2005). Central odontogenic fibroma: a case report. Med Oral Patol Oral Cir Bucal.

[14] Dunlap CL, Barker BF (1984). Central odontogenic fibroma of the WHO type. Oral Surg Oral Med Oral Pathol.

[15] Alawi F, Quinn P (2004). Atypical central odontogenic fibroma recu¬rring as ameloblastoma. Oral Surg Oral Pathol Oral Radiol Endod.

